# Emmental Cheese Environment Enhances *Propionibacterium freudenreichii* Stress Tolerance

**DOI:** 10.1371/journal.pone.0135780

**Published:** 2015-08-14

**Authors:** Valérie Gagnaire, Julien Jardin, Houem Rabah, Valérie Briard-Bion, Gwénaël Jan

**Affiliations:** 1 INRA, UMR1253 STLO, Science et Technologie du Lait et de l'Œuf, Rennes, France; 2 AGROCAMPUS OUEST, UMR1253 STLO, Rennes, France; Agricultural University of Athens, GREECE

## Abstract

Dairy propionibacteria are actinomycetales found in various fermented food products. The main species, *Propionibacterium freudenreichii*, is generally recognized as safe and used both as probiotic and as cheese starter. Its probiotic efficacy tightly depends on its tolerance towards digestive stresses, which can be largely modulated by the ingested delivery vehicle. Indeed, tolerance of this bacterium is enhanced when it is consumed within a fermented dairy product, compared to a dried probiotic preparation. We investigated both stress tolerance and protein neosynthesis upon growth in i) chemically defined or ii) aqueous phase of Emmental cheeses. Although the same final population level was reached in both media, a slower growth and an enhanced survival of CIRM BIA 1 strain of *P*. *freudenreichii* subsp. *shermanii* was observed in Emmental juice, compared to chemically defined medium. This was accompanied by differences in substrates used and products released as well as overexpression of various early stress adaptation proteins in Emmental juice, compared to chemically defined medium, implied in protein folding, in aspartate catabolism, in biosynthesis of valine, leucine and isoleucine, in pyruvate metabolism in citrate cycle, in the propionate metabolism, as well as in oxidoreductases. All these changes led to a higher digestive stress tolerance after growth in Emmental juice. Mechanisms of stress adaptation were induced in this environment, in accordance with enhanced survival. This opens perspectives for the use of hard and semi-hard cheeses as delivery vehicle for probiotics with enhanced efficacy.

## Introduction

*Propionibacterium freudenreichii* is a beneficial bacterium which belongs to the actinomycetales order, a group of gram-positive bacteria known for their prolific production of various valuable metabolites [1]. *P*. *freudenreichii*, originally present in raw milk, is currently added as a starter in the production of hard cheeses such as Emmental. Indeed, it is responsible for the specific organoleptic properties of such cheeses, via the production of short chain fatty acids (SCFAs) and of esters, and for its opening, i.e. formation of holes via the production of carbon dioxide in the cheese wheel [2]. The technological process of Emmental cheese consists of a succession of abiotic stresses including cooking, acidification, stirring, moulding, salting and ripening in rooms at different temperatures [3]. However, *P*. *freudenreichii* adapts, survives, and reaches elevated population above 10^9^ live bacteria per g of ripened cheese, while lactic acid bacteria, which first grow in cheese and perform acidification during cheese making, undergo massive cell lysis thereafter [4]. This evidences the ability to adapt to a succession of thermal, osmotic and other stresses during the cheese process. The average consumption of Emmental cheese in France, close to 30 g per day per person, constitutes a massive uptake of *P*. *freudenreichii*, 10^10^ live bacteria per day per person. Its impact on human health is thus a relevant issue. Accordingly, its probiotic abilities are also considered [5] including modulation of gut microbiota [6], gut inflammation [7,8] and proliferation/apoptosis balance [9]. Probiotic food supplements containing *P*. *freudenreichii* are commercially available [5].

*P*. *freudenreichii* probiotic potentialities are linked to its peculiar metabolic activity. It can modulate important parameters at the level of the gut, thanks to the release of beneficial metabolites previously described as nutraceuticals [10]. Its obligatory homofermentative metabolism, the propionic fermentation, relying on the Wood & Werkman cycle, leads to the production of the short chain fatty acids (SCFAs) acetate and propionate, regardless of the carbon source used. In accordance with the largely reported beneficial effects of SCFAs [11], *P*. *freudenreichii* metabolites were indeed shown to modulate the proliferation/apoptosis balance in the context of carcinogenesis, both *in vitro* [12,13] and *in vivo* [9]. Its metabolites also comprise 1,4-dihydroxy-2-naphtoic acid (DHNA), an intermediate in the synthesis of vitamin K, which was shown to enhance the colon bifidobacteria population in humans [14] and to modulate gut inflammation *in vivo* [15,16]. Such immunomodulatory effect is further enhanced by the production of surface extractible proteins [7,17]. To exert positive effects related to these beneficial metabolites, *P*. *freudenreichii* must reach the colon alive and metabolically active.

Hence, maintaining both survival and activity of this probiotic is a crucial bottleneck in its application. For this probiotic bacterium, and for others, the physiological state at the time of ingestion constitutes a crucial parameter of efficacy. *In vitro*, polymer encapsulation, or incorporation in a fermented dairy product, was shown to confer protection of *P*. *freudenreichii* towards acid and bile salts stress, and the combination thereof [18]. Accordingly, consumption of a yogurt containing *P*. *freudenreichii* led to enhanced survival and activity [19], compared to consumption of the same strain in a freeze-dried food supplement [20]. Emmental cheese also confers *P*. *freudenreichii* enhanced digestive stress tolerance [21]. Fermented dairy products, including fermented milks and Emmental cheese, were shown to provide dairy propionibacteria with elevated stress tolerance and stable populations, without lysis. However, the mechanisms leading to enhanced stress survival as a result of inclusion in a cheese are still unknown. Tolerance might be conferred by the protective and buffering effect of the cheese matrix. Indeed, lipids and proteins present in cheese may have a buffering effect towards acidic secretions, bile salts and hydrolytic enzymes. However, adaptation to the cheese environment, in terms of physico-chemical properties (pH, osmolarity) may also trigger expression of defense molecular mechanisms. The objective of this study was to identify stress tolerance mechanisms triggered during growth within the cheese environment. To address this question, the same *P*. *freudenreichii* strain, CIRM BIA1, was cultured either in laboratory chemically defined medium (CdM) [22], or in Emmental cheese aqueous phase also named Emmental juice (EJ). This juice was extracted from the cheese by hydraulic pressing, without dilution of the aqueous phase by any solvent or additional water, at the beginning of warm room ripening, after growth of lactic acid bacteria, when propionibacteria usually start to grow [23]. This medium contains the carbon and nitrogen sources essential for the propionibacteria growth, as well as other metabolites, moderately acid pH conditions and ionic environment encountered in cheese [24]. We investigated modulation of *P*. *freudenreichii* CIRM BIA1, which is the type strain, isolated from Emmental, and whose genome is sequenced [25]. In this strain, we investigated metabolic activities (consumption of substrates, production of metabolites) and stress adaptation in the cheese aqueous phase, *versus* the CdM.

This study reveals adaptation to the substrates present in cheese, enhanced survival in stationary phase, enhanced digestive stress tolerance and enhanced expression of key stress adaptation proteins, in the cheese aqueous phase. This work opens new perspectives for the development of optimized fermented functional food products, fa voring the probiotic potential of selected bacterial strains.

## Materials and Methods

### Micro-organisms and growth conditions

The CIRM BIA 1 strain of *P*. *freudenreichii* subsp. *shermanii* used in this study was kindly provided by the international microbial resource center CIRM BIA (INRA, Rennes, France). It was cultured in a chemically defined medium (CdM) [22] containing, per litre: 12.8 g sodium lactate, 0.6 g KH_2_PO_4_, 0.4 g potassium acetate, 50 mg MgSO_4_.7H_2_O, 5 mg MnSO_4_.4H_2_O, 2.5 mg FeSO_4_.7H_2_O, 2.5 mg CuSO_4_, 2.5 mg NaCl, 0.25 mg cobalt acetate, 15 μg ZnSO_4_, 1 μg H_3_BO_3_, 1 μg Na_2_MoO_4_, 50 μg thiamine, 100 μg pyridoxal, 50 μg calcium pantothenate, 50 μg riboflavine, 100 μg nicotinamide, 10 μg p-aminobenzoic acid, 4 μg biotine, 20 μg folic acid, 2 μg cyanocobalamine. Aminoacids were supplied at the following concentrations (L^-1^): 50 mg L-Ala, 160 mg L-Arg, 150 mg L-Asn, 250 mg L-Asp, 140 mg L-Cys, 80 mg Gly, 190 mg L-Glu, 150 mg L-Gln, 100 mg L-His, 180 mg L-Ile, 300 mg L-Leu, 220 mg L-Lys, 60 mg DL-Met, 460 mg L-Pro, 170 mg L-Phe, 180 mg L-Ser, 150 mg L-Thr, 50 mg L-Trp, 60 mg L-Tyr and 480 mg DL-Val. Five milligrams each of the bases adenine, guanine, uracile and xanthine were also added. The pH of the medium was adjusted to 7.0 prior to filter-sterilization (0.22 μm, Sartorius, Goettingen, Germany). Growth was carried out at 24°C without shaking under anaerobiosis (Anaerocult, Merck KGaA, Darmstadt, Germany). Growth curves were established by monitoring CFUs (Colony Forming Units, i.e. serial dilution and plate counting in YELA (Yeast Extract Lactate Agar medium) as described previously [22,26]. Propionibacteria physiological status was evaluated in exponential and stationary phase using live/dead staining (Invitrogen, Carlsbad, USA). Briefly, bacteria were centrifuged, resuspended in phosphate buffer saline (PBS) and doubled-stained with propidium iodide and SYTO 9 according to the instructions of Live Dead Light kit manufacturer’s instructions, prior to observation under an Optiphot fluorescence microscope (Nikon, Champigny sur Marne, France).

As an alternative, Emmental cheese aqueous phase was used as a growth medium and the duration of growth monitoring adapted under the same growth incubation conditions than in CdM. Three independent cultures were performed on the same batch of EJ or of CdM.

### Emmental cheese aqueous phase extraction

The aqueous phase of Emmental cheese (called Emmental juice hereafter, EJ) was extracted from several 2–2.5 kg cheese sectors from different wheels when the cheese wheels entered in the warm room that corresponds to the beginning of propionibacteria growth period. Cheese sectors were frozen at -20°C until use. Cheese sectors were then thawed for 15–20h at 4°C prior to the cheese aqueous phase extraction by using a hydraulic press according to Salvat-Brunaud et al [23]. The EJ (~150 ml) was successively filtered through Whatman paper 541 (Prolabo, Bruchet Dano, Rennes, France) then through 1.2- and 0.45-μm pore size cellulose acetate membrane filters (Sartorius, Palaiseau, France). After filter sterilization, EJ was stored at -20°C until use. The juice thus obtained was an average of different cheeses and the overall juice composition was in agreement and in range of the juices extracted in previous studies [4,23].

### Biochemical analysis of culture supernatants

*P*. *freudenreichii* CIRM BIA 1 was cultured in CdM or in EJ at 24°C as described above. At different culture times indicated in the corresponding result section, culture supernatants from EJ and CdM were prepared by centrifugation (8000 × g, 4°C, 10 min) to remove bacterial cells followed by filter-sterilization (0.22 μm, Sartorius, Goettingen, Germany) and conserved at -20°C until use. Both organic acid and free amino acid composition were determined as follows.

#### Organic acids analysis

The sterilized culture supernatants were half-diluted with 0.02 N H_2_SO_4_ and centrifuged at 8,000 × *g* for 30-min at 4°C to discard the protein pellet, the supernatant was filtrated and sugar and organic acids separated by high performance liquid chromatography (HPLC) by using an Aminex A-6 ion exchange column (Dionex, Sunnyvale, CA) at 55°C with 0.01 N H_2_SO_4_ as the eluent at a flow rate of 1 mL min^-1^. Both UV (210 nm) and refractometric detectors were used.

#### Free amino acid composition

The free-amino acid content of the sterilized culture supernatants was determined after deproteinization, first, to 1ml of samples were added 10 μl trifluoroacetic acid (Sigma-Aldrich). The acidified samples were shaken 15 s and centrifuged 1000 g for 10 min at 4°C and second, proteins were precipitated by adding 30 mg sulfosalicylic acid (Merck-Eurolab, Grosseron S.A., Saint Herblain, France) to the first supernatant, shaken for 15 s, and incubated for 1 h at 4°C. The samples were centrifuged at 8,000 × *g* for 15 min at 4°C. The resulting final supernatants were filtered through a 0.45-μm-pore-size membrane (Sartorius, Palaiseau, France), and the filtrate was diluted with a 0.2 mol.L^-1^ lithium citrate buffer (pH 2.2) to appropriate concentration. The diluted filtrates were injected onto a cation-exchange column of an automatic amino acid analyzer Biochrom 30 Plus (Biochrom Ltd, Cambridge, UK—SERLABO Technologies, Trappes, France) as first described by Spackman et al [27] using lithium citrate buffer for elution and a color reaction with ninhydrin at the column outlet for amino acid quantitation at dual wavelength of 570 nm and 440 nm.

### Osmolarity

The osmolarity of the CdM and EJ was measured in triplicate with a freezing-point osmometer (Osmomat 030-D, Gonotec, Berlin, Germany).

### Stress challenges

#### Acid challenge

Propionibacteria were harvested by centrifugation (8,000 × *g*, 24°C, 10 min) and recovered in an equal volume of CdM at pH 2.0 as described previously [22]. Viable-cell counts were determined after 30 min of acid challenge. Samples were diluted in peptone water (0.1% peptic digest of meat, Biokar Diagnostics, Beauvais, France), pH 7.0, containing 0.9% NaCl and poured in YEL medium (Yeast Extract Lactate) containing 1.5% agar for maximal recovery. CFU were determined after 6 days of anaerobic incubation at 30°C. Three independent cultures were performed from the same batch of CdM or of EJ.

#### Bile salts challenge

Propionibacteria were harvested by centrifugation (8000 × *g*, room temperature, 10 min) and recovered in an equal volume of CdM at pH 7.0 containing 1.0 g l^-1^ bile salts as described previously [3]. Viable-cell counts were determined after 30 min of bile salts challenge, by CFU counting as described in the micro-organisms and growth conditions section.

### Statistical analyses

Analyses of variance (ANOVA) were performed with the significance level set at *P*≤0.05, either using growth medium as one factor with FactoMineR, an R package [28], or two factors using medium culture and stage of growth. Newman Keuls test was performed as post ANOVA analysis.

### Proteomic studies

#### Radioactive labeling and whole-cell protein extraction

Propionibacteria were labeled essentially as described previously [22]. An exponential phase preculture in CdM was used to inoculate (1/100) either fresh CdM or EJ, both added with 500 μCi of [^35^S]methionine / cysteine protein labeling mix (175 Ci/mmol, ICN Pharmaceuticals, Orsay, France). Labeling thus occurred continuously between inoculation and harvesting at different times indicated in the corresponding result section. At harvesting, incorporation was stopped by rapidly washing the bacteria in PBS. Whole cell SDS extracts were immediately prepared according to a procedure modified from one previously described [29]. Washed bacteria were harvested by centrifugation (10 000 × *g*, 10 min, 4°C) prior to resuspension in SDS lysis buffer (50 mM Tris-HCl pH 7.5, 0.3% SDS, 200 mM DTT) to a final OD_650_ of 20. After 3 freeze/thaw cycles, bacteria were broken by sonication using a Vibra Cell sonicator (Bioblock Scientific, Illkirch, France) equipped with a tapered microtip (4 bursts of 1 min at 1 min intervals, output 2.5). Insoluble materials were removed by centrifugation (10 000 × *g*, 10 min, room temperature). The resulting whole-cell protein SDS extract was used for proteomic investigations.

#### Two-dimensional electrophoresis

Proteins were precipitated and washed using the 2-D Clean-Up kit (GE Healthcare Bio-Sciences AB). Air-dried protein pellets were solubilized in sample solution containing 7 M urea, 2 M thiourea, 25 mM DTT, 4% 3-[(3-Cholamidopropyl)dimethylammonio]-1-propane-sulfonate (CHAPS) and 2% IPG-Buffer (GE Healthcare Bio-Sciences AB, Upsalla, Sweden). Equal amounts of radioactivity (10^6^ dpm) were loaded onto the gel in the first dimension. Isoelectric focusing was carried out using pH 4 to 7 Immobiline Dry Strips on a Multiphor II electrophoresis system (GE Healthcare Bio-Sciences AB). Two-dimensional separation was performed according to our standardized procedure [22].

#### Image analysis

Radiolabeled gels were dried onto filter paper under vacuum. The radioactivity in the dried gels was detected using a Storm Phosphorimager (GE Healthcare Bio-Sciences AB). Non-radioactive gels were stained using Bio-Safe Coomassie G-250 (Bio-Rad laboratories, USA) prior to scanning on an ImageScanner III (GE Healthcare Bio-Sciences AB). Molecular weights and isoelectric points were calibrated by co-migrating standards (GE Healthcare Bio-Sciences AB).

Image analysis, gel matching and quantification of the radioactivity in individual spots were performed using the Progenesis SameSpot software 3.1 (Nonlinear Dynamics Newcastle upon Tyne, UK). All gels were compared by grouping them in EJ and CdM subgroups and to compute fold and and p-values of all spots using one way ANOVA analysis and Principal Component Analysis (PCA) as described by Morris et al [30]. The spots of interest that have a protein expression significantly different between both groups, above 1.2 fold change were selected and spotted for further identification.

#### Protein identification by Nano-LC coupled on-line with tandem mass spectrometry

Gel pieces were washed with acetonitrile and ammonium bicarbonate solution, and then dried under vacuum in a SpeedVac concentrator (SVC100H-200; Savant, Thermo Fisher Scientific, Waltham, MA, USA). In-gel trypsin digestion was performed overnight at 37°C and stopped with spectrophotometric-grade trifluoroacetic acid (TFA) (Sigma-Aldrich) as described previously [29]. The supernatants containing peptides were then vacuum dried in Speed-Vac concentrator and stored at -20°C until mass spectrometry analysis.

Nano-LC-MS/MS analysis was performed using an on-line liquid chromatography tandem mass spectrometry (MS/MS) setup using a Dionex U3000-RSLC nano-LC system fitted to a QSTAR _XL_ (MDS SCIEX, Ontario, Canada) equipped with a nano-electrospray ion source (ESI) (Proxeon Biosystems A/S, Odense, Denmark). Samples were first concentrated on a PepMap 100 reverse-phase column (C18, 5 μm particle size, 300-μm inner diameter (i.d.) by 5 mm length) (Dionex, Amsterdam, The Netherlands). Peptides were separated on a reverse-phase PepMap 100 column (C18, 3 μm particle size, 75 μm i.d. by 150 mm length) (Dionex) at 35°C, using solvent A (2% (vol/vol) acetonitrile, 0.08% (vol/vol) formic acid, and 0.01% (vol/vol) TFA in deionized water) and solvent B (95% (vol/vol) acetonitrile, 0.08% (vol/vol) formic acid, and 0.01% (vol/vol) TFA in deionized water). A linear gradient from 10 to 40% of solvent B in 17 min was applied for the elution at a flow rate of 0.3 μL min^-1^. Eluted peptides were directly electrosprayed into the mass spectrometer operated in positive mode. A full continuous MS scan was carried out followed by three data-dependent MS/MS scans. Spectra were collected in the selected mass range 300 to 2 000 m/z for MS and 60 to 2 000 m/z for MS/MS spectra. 2^+^ to 4^+^ charged ions were considered for the MS/MS analysis when ion intensity was above 10 cps. The mass spectrometer was operated in data-dependent mode automatically switching between MS and MS/MS acquisition using Analyst QS 1.1 software. The instrument was calibrated by multipoint calibration using fragment ions that resulted from the collision-induced decomposition of a peptide from β-casein, β-CN (193–209).

To identify peptides, all data (MS and MS/MS) were submitted to X! Tandem using the X! Tandem pipeline developed by PAPPSO (Plateforme d'Analyse Protéomique de Paris Sud-Ouest (PAPPSO), INRA, Jouy-en-Josas, France, http://pappso.inra.fr).

The search was performed against a database composed of (i) a homemade database containing all the predicted proteins of the *P*. *freudenreichii* strain CIRM-BIA 1 used in this study and (ii) a portion of the UniProtKB database corresponding to *P*. *freudenreichii*.

Database search parameters were specified as follow: trypsin cleavage was used and the peptide mass tolerance was set to 0.2Da for both MS and MS/MS. Oxidation of methionine and deamidation of asparagine and glutamine residues were included as variable modifications. Protein identifications were automatically validated when they showed at least two unique peptides with an e-value below 0.05.

## Results

### Slower growth and enhanced survival of CIRM BIA 1 strain of *P*. *freudenreichii* subsp. *shermanii* in EJ compared to CdM

**Fig 1:**
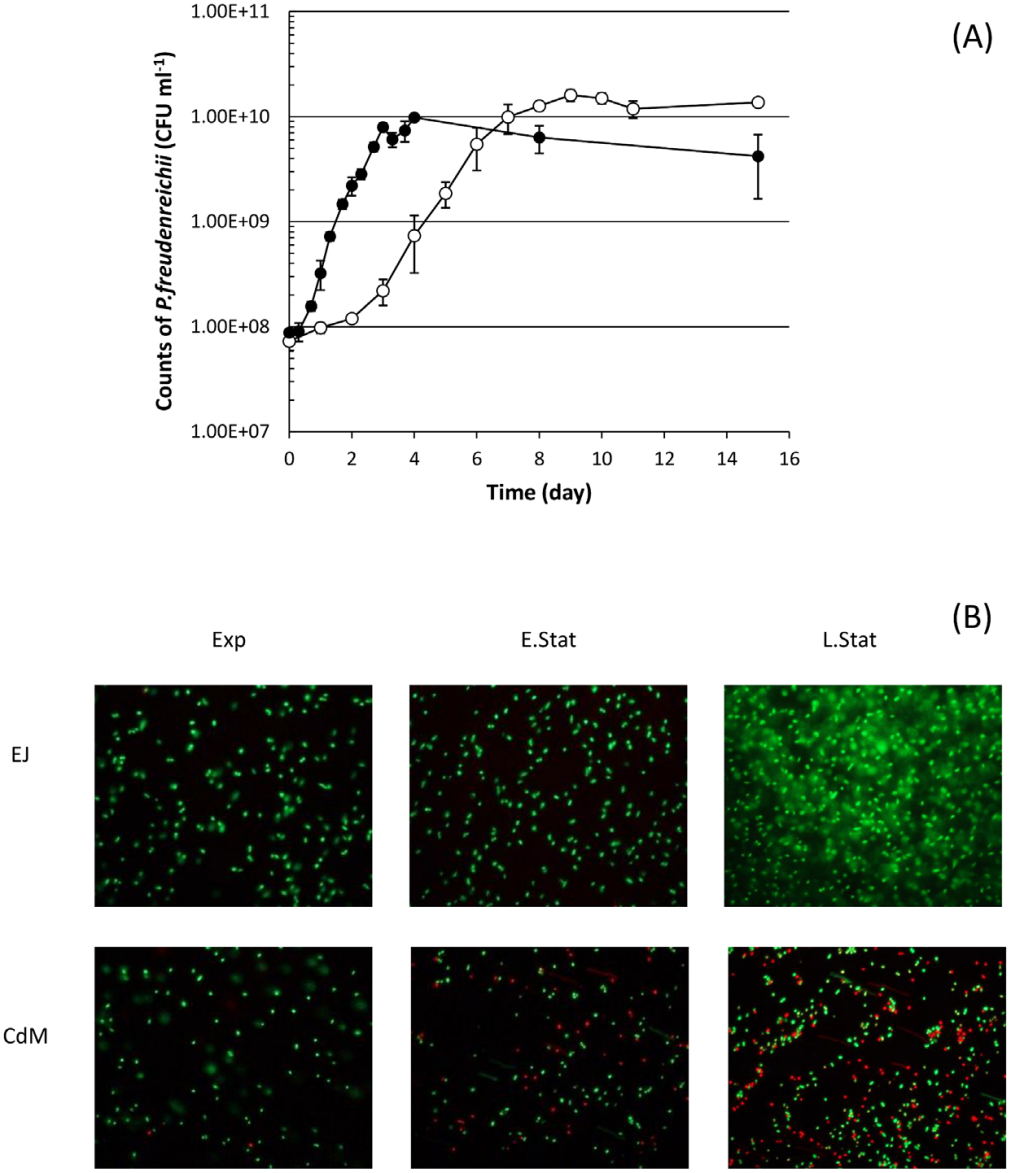
Growth and survival of *P*. *freudenreichii* subsp. *shermani* CIRM BIA 1 in EJ and CdM (A) growth curve and (B) Live/dead staining of the bacterial cells in the exponential (Exp), early stationary (E.Stat) and late stationary phase (L.Stat) of growth in CdM (**black dot**) and in EJ (**white dot**).

When comparing growth in EJ, *versus* in CdM, CIRM BIA 1 strain of *P*. *freudenreichii* subsp. s*hermani* reached similar maximum population level at the early stationary phase in both media, i.e. 1.60 x10^10^ CFU mL^-1^ and 9.83 x10^9^ CFU mL^-1^ for EJ and CdM respectively (Fig 1). However, the growth rate was greatly reduced in EJ compared to CdM since the doubling time was twice higher in EJ (22.58 h) than in CdM (10.34 h). The slower growth in the CdM compared to a previous study on the same medium [22] was due to growth at 24°C instead of the optimal temperature of 30°C. In CdM, red fluorescent propionibacteria cells considered as dead were observed in early and late stationary phase in accordance with the decrease in cultivability observed after 4 days of culture (Fig 1B). By contrast very few dead cells were observed in EJ (Fig 1B).

### Differences in substrates used and products released by *P*. *freudenreichii* subsp. *shermani* CIRM BIA 1 between EJ and CdM

**Fig 2:**
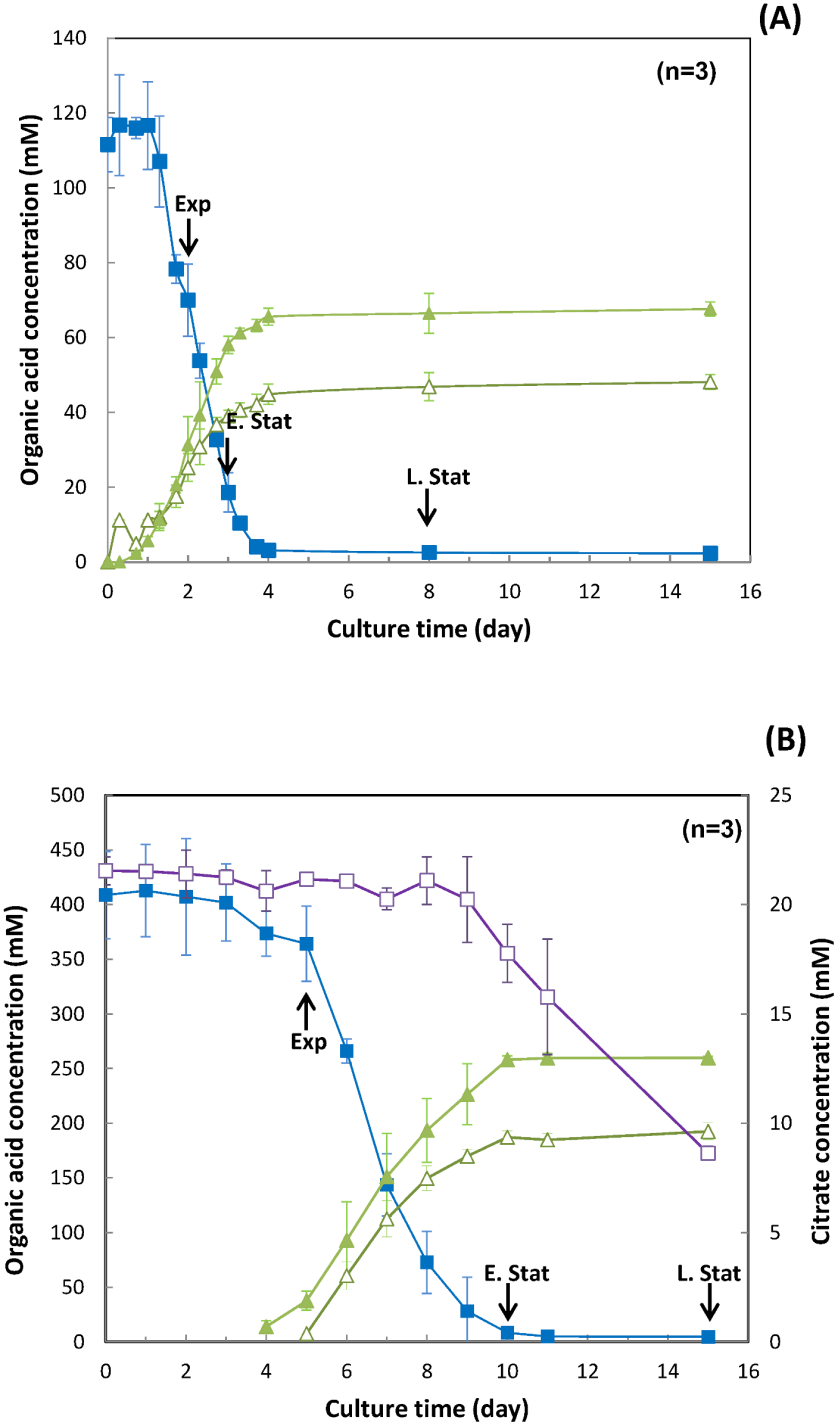
Degradation and production of various organic acids during growth of *P*. *freudenreichii* subsp. *shermani* CIRM BIA 1 in (A) CdM and (B) EJ. blue square lactic acid; white and green triangle acetic acid, green triangle propionic acid and purple square citric acid. The arrows indicate the main growth stages corresponding to each incubation medium: exponential (Exp), early stationary (E.Stat) and late stationary phase (L.Stat). Lactate was almost entirely consumed in both media with less than 3% left at the end of culture time. The plateau in CdM was observed in late stationary phase (98% lactate degradation). In the case of EJ, citrate was present concomitantly with lactate and used by *P*. *freudenreichii* subsp. *shermani* CIRM BIA 1 as a carbon source after lactate was exhausted *i*.*e*., at early stationary phase.

**Fig 3:**
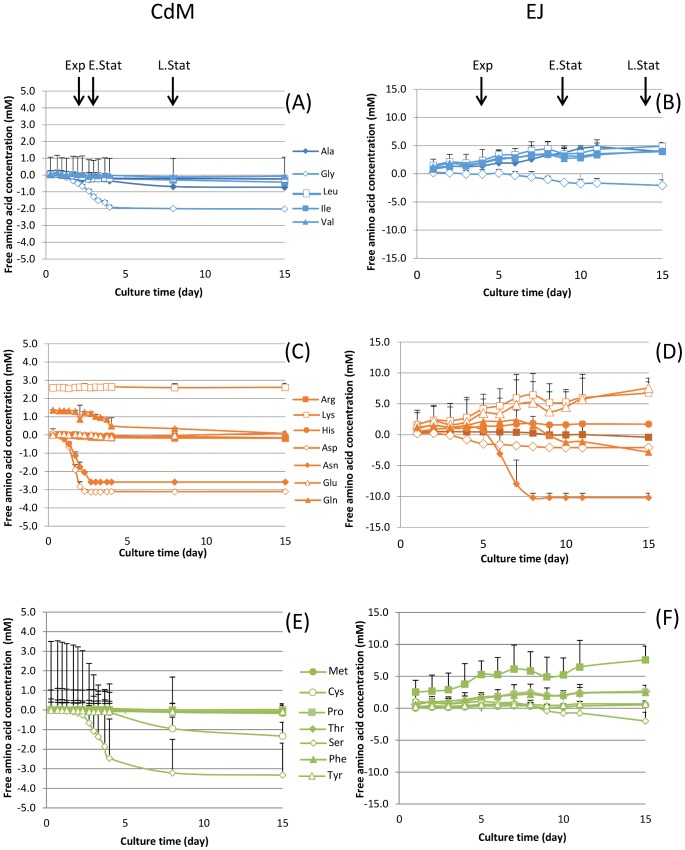
Relative change in free amino acid concentration during growth of *P*. *freudenreichii* subsp. *shermani* CIRM BIA 1 in CdM (A, C and E) and EJ (B, D and F). (A and B) Alliphatic amino acids; (C and D) basic, acidic + amide amino acids; (E and F) Aromatic, hydroxyl and sulfur-containing amino acids. The arrows indicate the main growth phases corresponding to each incubation medium: exponential (Exp), early stationary (E.Stat) and late stationary phase (L.Stat).

Organic acids (Fig 2) and free amino acids (Fig 3) were specifically monitored in both EJ and CdM during propionibacteria cell growth. The osmolarity was 1368 ± 14 mosmol L^-1^ in EJ and 367 ± 1 mosmol L^-1^ in CdM.

The lactate content was 120 mM and 400 mM in CdM and in EJ respectively (Fig 2). The higher content observed in EJ was in agreement with the initial lactose content in milk that is used by lactic acid bacteria as a first carbohydrate substrate and converted to lactate during cheese making, which is in turn used by *P*. *freudenreichii* subsp. *shermani* CIRM BIA 1.

Regarding the amino acids (Fig 3), the initial values of amino acid concentration in CdM or EJ are presented in Table 1. Most of the amino acids were consumed in CdM and some of them entirely as glycine, aspartic acid, asparagine, cysteine and serine. Indeed, alanine glycine, aspartate, asparagine, glutamine and serine were mainly consumed in CdM concomitantly with lactate. Asparagine, aspartate, glycine and serine were also the most heavily consumed compared to the other free amino acids in EJ. In contrast to CdM, most of the other amino acids increased with time.

**Table 1:** Initial composition in free amino acids of the EJ and the CdM.

Free Amino acid name	CdM		EJ	
	Mean value (mM)	SD	Mean value(mM)	SD
Ala	0.73	0.01	5.86	0.22
Gly	2.06	0.06	4.68	0.36
Leu	3.39	0.93	13.48	0.98
Ile	2.93	0.12	5.20	0.41
Val	5.27	3.85	11.22	0.96
Arg	1.59	0.04	2.39	0.08
Asp	3.13	0.06	2.16	0.22
Asn	2.58	0.04	9.88	0.71
Glu	1.81	0.02	20.72	1.48
Gln	1.35	0.03	9.28	0.56
His	1.12	0.07	1.80	0.10
Lys	2.57	0.06	14.80	1.50
Met	1.09	0.24	2.90	0.41
Cys	2.01	0.52	0.00	1.26
Phe	2.10	0.04	3.26	0.17
Pro	0.00	0.00	13.05	1.47
Thr	2.73	0.18	6.32	0.52
Tyr	0.45	0.19	2.03	0.07
Ser	2.26	1.63	5.63	0.45

The concentrations in free amino acids were measured in triplicate.

CdM: Chemically defined medium;

EJ: Emmental cheese aqueous phase

### Overexpression of early stress adaptation proteins in EJ compared to CdM

**Fig 4:**
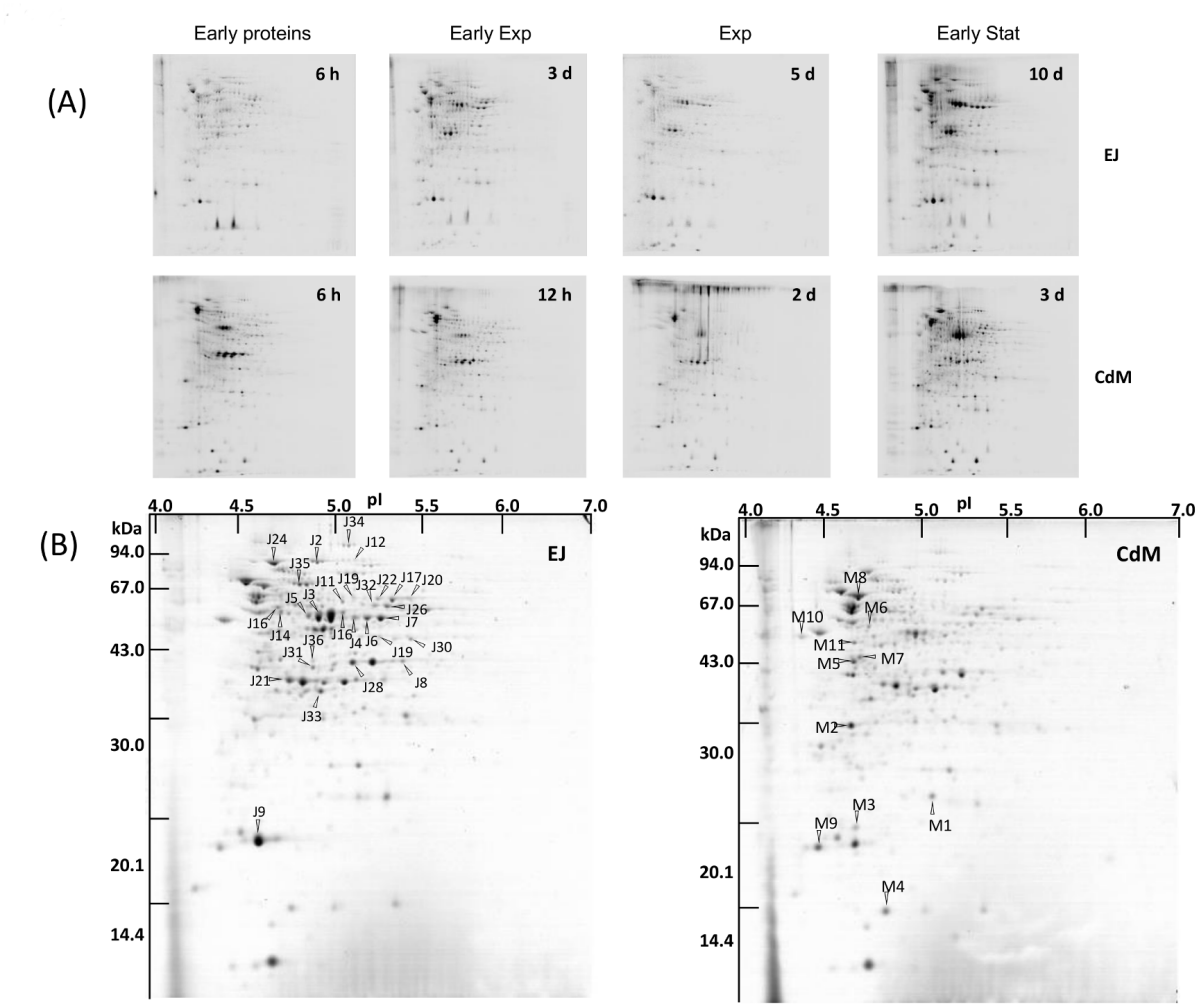
Two-dimensional analysis of protein expression during growth of *P*. *freudenreichii* subsp. *shermani* CIRM BIA 1 in EJ and in CdM. (A) Neosynthesized proteins were radiolabeled with ^35^S amino acids in both media during indicated duration: lag phase (early), the beginning of the exponential phase (early exponential), the exponential and early stationary phases. (B) Cellular proteins accumulated during growth were Coomassie blue stained.

To determine which proteins are neosynthezised in CdM and EJ, a proteomic study was performed (Fig 4).

Proteins which were differentially expressed in EJ or in CdM were spotted from 2D gels and identified by nano LC coupled on-line with tandem mass spectrometry. The identified proteins are shown in Table 2.

**Table 2:** Proteins differentially expressed after growth of *P*. *freudenreichii* subsp. *shermani* CIRM BIA 1 in EJ and in CdM and identified by on line coupling NanoLC-ESI-Q-TOF tandem mass spectrometry.

Spot	LocusTag	Description	Name	pI	Molecular mass (Da)	Fold change*	Anova (p)	Coverage (%)	log(e-value)**
**Proteins differentially expressed after growth in CdM**									
		*Membrane bioenergetics*							
M10	PFCIRM1_03505	Coenzyme F420-dependent N5,N10-methylene tetrahydromethanopterin reductase	mer	4.1	53280	2.0	0.954	23	-21.59
M11	PFCIRM1_01295	Cytochrome P450	cypA	4.5	51097	2.5	0.645	50	-57.83
		*Metabolism of nucleotides*							
M7	PFCIRM1_01495	Dihydroorotate dehydrogenase		4.5	45527	1.7	0.513	41	-50.77
M9	ND	ND		4.2	18491	1.9	0.874		
		*Protein folding*							
M3	PFCIRM1_04380	Peptidyl-prolyl cis-trans isomerase A	cypB	4.5	19832	2.9	0.152	38	-13.36
M4	PFCIRM1_05325	10 kDa chaperonin 1	groES1	4.7	14269	1.6	0.433	50	-12.12
M8	PFCIRM1_06910	Chaperone protein	dnaK 1	4.5	68960	1.6	0.189	50	-90.77
		*Protein modification*							
M5	PFCIRM1_11190	Thioredoxin	trxA3	4.5	44252	1.7	0.664	36	-24.92
		*Transport/binding of amino-acids*							
M6	PFCIRM1_10910	binding protein of oligopeptide ABC transporter		4.6	57118	2.8	0.021	53	-71.75
		*Unknown function*							
M1	PFCIRM1_01040	YceI family protein		5.1	22894	3.2	0.001	66	-39.59
M2	PFCIRM1_08890	Hypothetical protein		4.5	30264	1.9	0.024	65	-20.81
**Proteins differentially expressed after growth in EJ**									
		*Main glycolytic pathways*							
J16	PFCIRM1_10210	Glucose-6-phosphate isomerase	pgi	5.1	64419	1.9	0.350	26	-39.16
J28	PFCIRM1_04055	Glyceraldehyde-3-phosphate dehydrogenase	gap	5.2	42009	1.5	0.027	34	-31.55
J33	PFCIRM1_02085	Fructose-bisphosphate aldolase class I	fba2	5.0	36818	1.9	0.001	35	-37.40
		*Membrane bioenergetics*							
J14	PFCIRM1_06010	NADH-quinone oxidoreductase chain D	nuoD	4.7	59828	2.0	0.141	49	-73.36
J18	PFCIRM1_05265	FAD-dependent pyridine nucleotide-disulphide oxidoreductase		5.6	64043	1.6	0.406	30	-39.33
J34	PFREUD_01840	Pyruvate synthase/Pyruvate-flavodoxin oxidoreductase	nifJ1	5.2	123434	1.7	0.978	24	-76.44
		*Metabolism of aminoacids*							
J1	PFCIRM119_00645	Aspartate ammonia-lyase	aspA2	5.1	60280	6.2	0.012	30	-30.01
J3	PFREUD_16330	Aspartate ammonia-lyase	aspA2	5.0	58323	6.2	0.001	47	-57.43
J5	PFCIRM1_00205	Argininosuccinate synthase	argG	4.9	59753	5.4	0.032	23	-19.35
J10	PFCIRM119_02565	L-aspartate oxidase	nadB2	5.3	62613	2.2	0.145	37	-49.57
J11	PFCIRM119_00645	Aspartate ammonia-lyase	aspA2	5.0	59226	2.5	0.002	51	-57.04
J19	PFCIRM1_10665	4-aminobutyrate aminotransferase	gabT	5.4	51022	2.3	0.020	27	-42.13
J31	PFCIRM1_04705	Ketol-acid reductoisomerase	ilvC	4.9	42097	1.8	0.456	20	-25.28
J36	PFCIRM1_02920	Branched-chain amino acid aminotransferase	ilvE	4.9	43460	1.6	0.011	29	-22.19
		*Metabolism of carbohydrates*							
J15	PFCIRM1_03315	Phosphate acetyltransferase	pta	4.6	60204	1.8	0.064	49	-84.12
J21	PFCIRM1_08810	Malate dehydrogenase	mdh	4.7	39326	1.7	0.797	28	-24.36
J24	PFCIRM1_01390	Pyruvate phosphate dikinase	ppdk	4.6	90146	1.5	0.852	43	-121.11
J30	PFCIRM1_03320	Acetate kinase	ackA	5.6	48688	1.7	0.258	43	-33.17
		*Protein degradation*							
J4	PFCIRM1_01700	PepP Xaa-Pro aminopeptidase I	pepP	5.2	58323	4.2	0.010	29	-31.79
		*Protein folding*							
J2	PFREUD_19250	Chaperone clpB 1	clpB 1	4.9	93417	3.0	0.054	42	-99.21
J9	PFCIRM1_10645	Heat shock protein 20 2	hsp20 2	4.5	18705	1.6	0.056	58	-36.12
J12	PFCIRM1_07785	clpC chaperone	clpC	5.1	94196	3.4	0.527	29	-65.64
		*Protein synthesis*							
J13	PFCIRM1_07260	Arginyl-tRNA synthetase	argS	5.2	65774	1.7	0.324	24	-41.70
		*Specific carbohydrate metabolic pathway*							
J17	PFCIRM1_09655	Methylmalonyl-CoA carboxytransferase 12S subunit	mmdA	5.5	63742	1.8	0.234	43	-71.52
J20	PFCIRM1_09655	Methylmalonyl-CoA carboxytransferase 12S subunit	mmdA	5.6	64043	6.1	0.394	33	-43.46
J22	PFCIRM1_09655	Methylmalonyl-CoA carboxytransferase 12S subunit	mmdA	5.4	64269	2.0	0.035	29	-60.32
J26	PFCIRM1_09650	Methylmalonyl-CoA carboxytransferase 5S subunit		5.4	61785	2.2	0.912	35	-35.55
J32	PFCIRM1_09650	Methylmalonyl-CoA carboxytransferase 5S subunit		5.3	61935	2.0	0.800	14	-18.42
J35	PFCIRM1_02425	Methylmalonyl-CoA mutase small subunit	mutA	4.8	74412	2.1	0.256	40	-61.82
		*TCA cycle*							
J6	PFCIRM1_03875	Fumarate hydratase, class-II	fumC	5.3	58022	2.9	0.075	21	-20.10
J7	PFCIRM1_03875	Fumarate hydratase, class-II	fumC	5.4	57720	2.6	0.029	30	-36.69
		*Unknown function*							
J8	PFCIRM1_02805	zinc-binding dehydrogenase		5.6	41877	3.2	0.005	28	-30.70

*: Fold changes correspond to the protein overexpressed with a minimum fold change of 1.2 in the CdM or EJ determined by image analysis

**: e-value is the number of times a given peptide score will be achieved by incorrect matches from a database search. Protein identifications were automatically validated when they showed at least two unique peptides with an e-value below 0.05 corresponding to log(e-value) < -1.30

First, cells were pulse labelled by incubating cells with ^35^S amino acids to monitor early neosynthesis of proteins during different duration of adaptation to both media (Fig 4A). Early adaptation proteins were clearly distinct when comparing both media. Important differences in the expression profiles persisted until stationary phase. This was confirmed by different accumulation of cellular proteins, as revealed by Coomassie staining at the end of growth in CdM and EJ (Fig 4B). Proteins that were differentially expressed with fold changes above 1.2 in CdM and in EJ were spotted (Fig 4B), identified by liquid chromatography coupled on-line with mass tandem spectrometry and reported in Table 2.

The proteins that were overexpressed in CdM, are involved in protein folding with the identification of three chaperone proteins (dnaK1, groES1 and cypB), in oxidoreduction reactions with cytochrome P450, thioredoxin and one reductase (mer) as well as in transport with one protein belonging to the ABC oligopeptide transporter and an enzyme implied in DNA synthesis.

In EJ, other chaperon proteins were overexpressed, clpB1 clpC and hsp20 as well as oxidoreductases. The main differences lied in the highly abundant proteins involved in aspartate catabolism (aspartate ammonia lyase that deaminates aspartate into fumarate [25]; L-aspartate oxidase, 4-aminobutyrate aminotransferase to transform succinate semi aldehyde into GABA), in biosynthesis of valine, leucine and isoleucine (ilv C and E proteins), in pyruvate metabolism (pta, mdh, ppdk and ackA), in citrate cycle and in the propionate metabolism with the well-known methyl-malonyl CoA carboxytransferase.

### Higher digestive stress tolerance after growth of *P*. *freudenreichii* subsp. *shermani* CIRM BIA 1 in EJ compared to CdM

**Fig 5:**
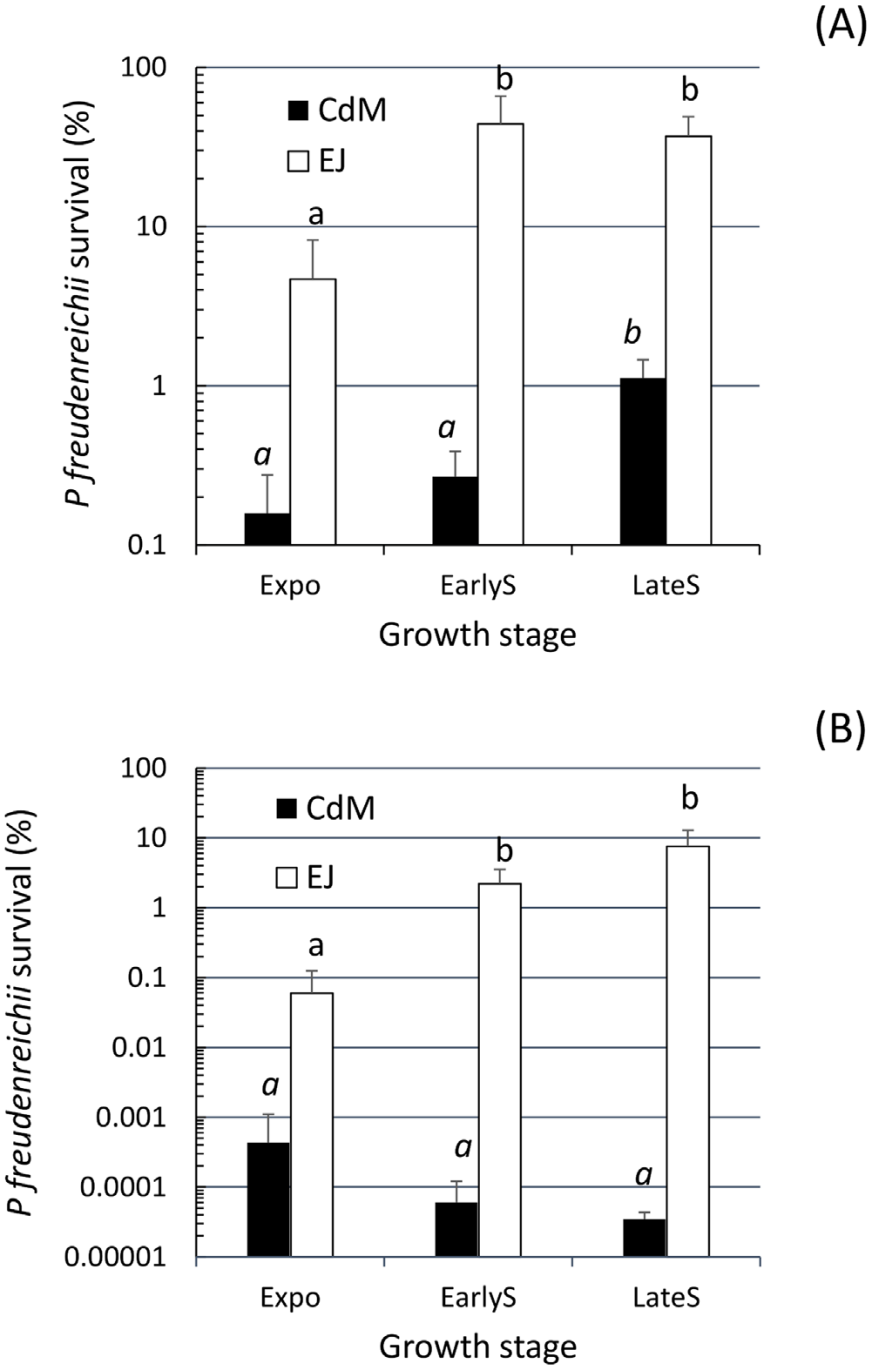
*P*. *freudenreichii* subsp. *shermani* CIRM BIA 1 tolerance to digestive stresses. Propionibacteria were harvested at different stages of growth in CdM black square or in EJ white square, prior to acid (A, pH 2) or bile salts (B, 1 g L^-1^) challenges. Surviving bacteria were then counted by CFU enumeration. Means with different lower case superscript letters (a-b) differ significantly (*P* < 0.05) in italic groups for CdM and in normal case groups for EJ.

Digestive stress tolerance was sought after growth of *P*. *freudenreichii* subsp. *shermani* CIRM-BIA1 in both media (Fig 5). Cells grown in CdM underwent significant severe mortality compared to EJ for both acid (Fig 5A) and bile salts (Fig 5B) challenge stresses at P<0.05.

For both culture media, the acid tolerance differed depending on the growth phase from which the cells were harvested prior to the acid challenge test. In CdM, there was an increase in survival rate from exponential to late stationary phase. In EJ, the maximum survival rate was already reached in the early stationary phase.

Regarding the bile salts challenge stress (Fig 5B), fewer cell survival was observed compared to the acid challenge stress for both culture media, with a maximum of 10% observed for EJ in late stationary phase while the percentage was lower than 0.001% for CdM. For both media, bile salts tolerance also depended on growth phase (Fig 5B). In CdM, tolerance decreased while in EJ it increased when the cells entered the stationary phase, showing significantly enhanced tolerance acquisition in this medium (P<0.05).

### Impact of osmoadaptation on *P*. *freudenreichii* subsp. *shermani* CIRM BIA 1 stress tolerance

The osmolarity of CdM was much lower than that of EJ, we thus investigated the role of osmolarity and osmoadaptation in tolerance acquisition by *P*. *freudenreichii* CIRM BIA 1. As can be seen in S1 Fig, addition of 0.45 M NaCl to the CdM, in order to reach the same osmolarity as in EJ (>1000 mosmol L^-1^), does in fact cause enhanced stress susceptibility towards the acid and bile salts. However, addition of 10 g of Yeast Extract L^-1^ of salted CdM restored stress tolerance.

## Discussion

Stress tolerance is a key characteristic for probiotic application of bacteria. For an optimal probiotic efficacy, they have to survive in the probiotic product during storage, in order to tolerate digestive stresses, including acid stress in the stomach and bile salts stress in the duodenum, to reach the colon alive. Indeed, the expected beneficial effects of probiotic bacteria generally depend on *in situ* synthesis of beneficial metabolites, such as short chain fatty acids and bifidogenic compounds, as in *P*. *freudenreichii* [5]. Previous studies showed that preadaptation can trigger high levels of tolerance in *P*. *freudenreichii* [3,22,29,31]. The conditions encountered within the probiotic delivery vehicle plays a key role in the probiotic efficacy. The cheese environment was shown to confer enhanced tolerance and survival compared to laboratory media [21]. We thus investigated growth and tolerance acquisition both within laboratory CdM and in EJ. EJ does not totally reflect prolonged residence in Emmental cheese, but rather allows growth with the substrates that *P*. *freudenreichii* uses when growing in a cheese. It has been developed to provide for propionibacteria growth and environment as similar as possible to the one prevailing in a cheese [23,32]. The EJ used in this study was prepared from different sectors of different Emmental wheels entering the warm room to represent an average of cheese aqueous phase at this stage.

CdM, previously developed to investigate molecular mechanisms in *P*. *freudenreichii* [22], contains all the necessary growth factors as well as lactate, propionibacteria preferred carbon source and neutral pH. In contrast,EJ extracted from cheese, triggers high salt osmotic up-shocks, has acid pH in range 5.4–5.7. mainly contains lactate (35–37 g kg^-1^ EJ, *i*.*e*. 380–410 mM), amino acids (22–30 g kg^-1^), sodium (316–430 mM), and calcium (171–200 mM) [32] in higher amount than in CdM, as well as other components such as citrate, peptides (22–30 g kg^-1^), other minor components already present in milk before cheese making [23,24,32]. EJ also contains bacterial metabolites including enzymes resulting from the initial growth of lactic acid bacteria released by lysis in cheese before extraction of EJ [24], that are not present in CdM.

In this study we clearly showed that *P*. *freudenreichii* subsp. *shermani* CIRM-BIA1 grew differently in CdM and in EJ although the same final cell population was reached in early stationary phase for both media (Fig 1). After using lactate in EJ, two other ways were used to supply energy to the cells: the aspartate/asparagine and the citrate metabolism. This was evidenced both at metabolic and protein levels (Fig 3 and Table 2).

Acetate and propionate were produced from lactate according to the well-known theoretical equation of Fitz [33], *i*.*e*., 3 moles of lactate are transformed into 1 mole of acetate, 2 moles of propionate and 1 mole of carbon dioxide (this latter not measured in our study). The amount of propionate produced in both media was in accordance with the equation. However, it was observed that the molar ratio of propionate to acetate was lower than the theoretical ratio since the effective ratio propionate:acetate was 1.34 in EJ and 1.40 in CdM. This was already observed in Swiss type cheeses due to concomitant production of acetate from other metabolic pathways, such as amino acid catabolism from aspartate, serine alanine and glycine [34–36]. Indeed, alanine, glycine, aspartate, asparagine, glutamine and serine were mainly used in CdM concomitantly with lactate. Asparagine, aspartate, glycine and serine were also the most heavily consumed compared to the other free amino acids in EJ and many proteins from the aspartate metabolism (aspartate ammonia-lyase, argininosuccinate synthase, L-aspartate oxidase) were overexpressed in EJ (Table 2). Notably, acetate and propionate production was still slightly increasing after lactate and aspartate exhaustion likely by using alanine and serine in agreement with results of Crow [34]. Branched-chain amino acids catabolism, showed by ketol-acid reductoisomerase and branched-chain amino acid aminotransferase, was overexpressed in EJ compared to CdM, in accordance with *P*. *freudenreichii* ability to produce high level of branched-chain volatile compounds in Swiss-type cheeses. Such enzymes are also implied in the synthesis of branched-chain fatty acids that derived from the catabolism of valine, leucine and isoleucine and that may help reinforcing the lipid membranes to cope with cold stress during cheese storage [35,36].

In contrast to CdM, most of the other amino acids increased with time in EJ, due to the presence of active peptidases [4,24]. The high content in glutamic acid, proline, lysine, leucine, valine and alanine in EJ before and during propionibacteria growth also reflected the composition of the casein–derived peptides thereof.

Enhanced stress tolerance, in this work, is the main physiological consequence of growth within EJ, compared to CdM. Indeed, enhanced survival under both acid and bile salts stress conditions was observed. Tolerance towards a specific stress (acid, bile salts, heat) is generally obtained by a homologous sub-lethal pre-treatment in *P*. *freudenreichii* [3,22,29,31], which is not the case here. General stress tolerance can also be triggered by starvation, as a result of entry into stationary phase in *P*. *freudenreichii* [18] and in other bacteria [37]. Accordingly, *P*. *freudenreichii* was shown here to gain stress tolerance upon entry into stationary phase (Fig 5). However, in our study, higher tolerance was observed in EJ, regardless of the growth phase. Even in exponential phase did EJ confer enhanced stress tolerance. The main stimulus responsible for this adaptation may be osmotic pressure. Indeed, EJ, mainly because of the salt added to cheese, revealed a higher osmolarity, 1368 ± 14 mosmol L^-1^ in EJ in agreement with results of Salvat–Brunaud et al [32] compared to 367 ± 1 mosmol L^-1^ in CdM. Osmotic stress was reported to trigger cross-protection to other stresses, including bile salts stress [38,39]. In our work, osmotic stress was shown to reduce stress tolerance in CdM while addition of yeast extract in salted CdM increased salt tolerance showing that externally provided compatible solutes, in conjunction with osmoadaptation, play a crucial role in stress tolerance, as described in other bacteria [38,40]. We suggest that such solutes may be provided either in higher concentration in EJ than in CdM + yeast extract or that EJ provides other potent osmoprotectants not present in the yeast extract. Indeed, milk and/or ripened cheeses are known to contain choline, betaine, proline, carnitine [41,42] and dimethyl-sulfonio-propionate known as preferential osmoprotectant of *P*. *freudenreichii* [43].

In accordance, the amino acids proline and glycine potent osmoprotectants used in the osmoprotectant transport system of *P*. *freudenreichii* subsp. *shermani* CIRM-BIA1 [25], were shown here to be available in EJ and not in CdM, throughout the growth and even during stationary phase. The primary role of osmoprotectant, whether they are taken up from the external medium (glycine betaine) or synthesized *de novo* intracellularly (trehalose), is to restore turgescent pressure as a result of intracellular accumulation, a crucial parameter for growth and division [44]. Another role is due to the “chaperone” effect of osmoprotectant, which protects macromolecules from salt-induced denaturation and favors normal cellular and molecular processes [44]. *P*. *freudenreichii* was reported to accumulate, as a result of osmoadaptation, high levels of glycine betaine, glutamate, trehalose and glycogen [35,43]. Such accumulated protectant molecules may well explain the observed enhanced survival, evidenced both by CFU counting and by physiological staining, after several days in stationary phase (Fig 1). According to this observed stress tolerance response, cellular accumulation of stress adaptation proteins was observed here upon growth in EJ. These proteins were previously identified in *P*. *freudenreichii* as involved in acid and/or bile salts adaptation. As an example, proteins involved in protein folding such as ClpB, ClpC and Hsp20 participate in acid and bile adaptation in *P*. *freudenreichii* and in other bacteria [29,45]. The same stands for aspartate ammonia-lyase, acid-and bile-inducible in *P*. *freudenreichii*, which takes part in amino acid catabolism and adaptation to acid conditions in enteric bacteria [46]. Moreover, the two subunits of the methylmalonyl-CoA carboxytransferase subunits, key enzyme of propionic fermentation, were shown to participate in *in vitro* acid and bile salts adaptation in *P*. *freudenreichii* [29] and to be expressed within the human digestive tract [19]. In accordance with enhanced stress tolerance in EJ, the cheese aqueous phase, propionibacteria are known to stay viable and cultivable in Emmental cheeses even after several months of ripening [47], while their viability constantly drops in CdM (data not shown).

As a conclusion, the growth conditions determine the stress tolerance, and thus the efficacy, of the probiotic *P*. *freudenreichii*. Growth within cheese is promising in this respect and imposes sublethal doses of stress triggering stress tolerance response. Thus, cheeses with well mastered microflora and physicochemical and technological parameters should be considered as efficient biofunctional food carriers [48].

## Supporting Information

S1 Fig *P*. *freudenreichii* subsp. *shermani* CIRM BIA 1 tolerance to digestive stresses in CdM and in CdM supplemented with 0.45 M NaCl or 0.45 M NaCl and 10 g L^-1^ yeast extract. Propionibacteria were harvested at stationary phase of growth in CdM prior to acid (A, pH 2) or bile salts (B, 1 g L^-1^) challenges as described in material and Method section. Surviving bacteria were then counted by CFU enumeration. Means with different lower case superscript letters (*a-c*) differ significantly (P < 0.05).
(TIF)
