# Supplementary figures and images for: Emmental Cheese Environment Enhances Propionibacterium freudenreichii Stress Tolerance
Source: PLoS One. 2015 Aug 14;10(8):e0135780. doi: 10.1371/journal.pone.0135780 (PMC4537189; doi:10.1371/journal.pone.0135780)

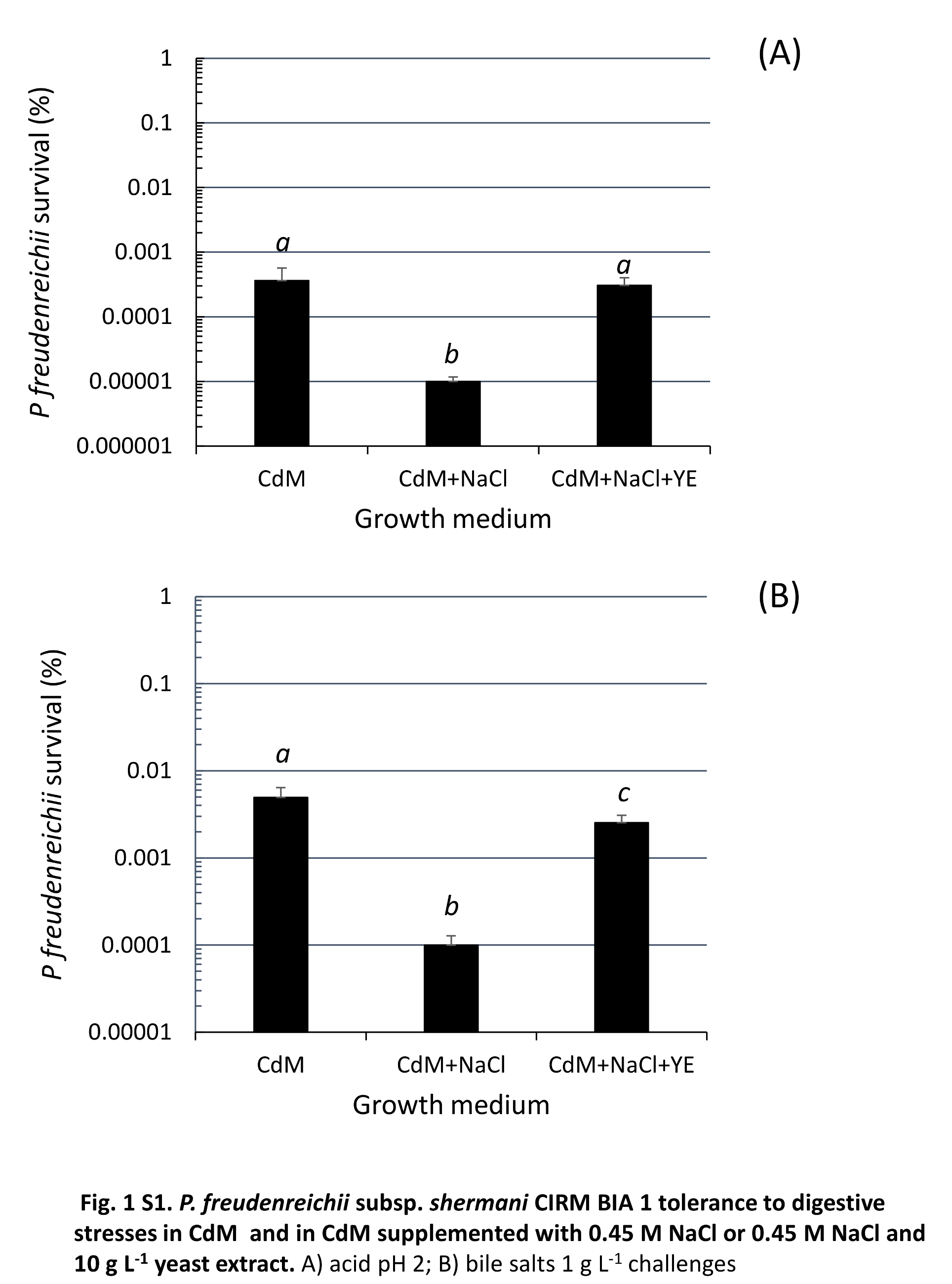

Supplement: S1 Fig — Propionibacteria were harvested at stationary phase of growth in CdM prior to acid (A, pH 2) or bile salts (B, 1 g L-1) challenges as described in material and Method section. Surviving bacteria were then counted by CFU enumeration. Means with different lower case superscript letters (a-c) differ significantly (P < 0.05). (TIF) [file pone.0135780.s001.tif]
